# Progress Toward Poliomyelitis Eradication — Pakistan, January 2022–June 2023

**DOI:** 10.15585/mmwr.mm7233a1

**Published:** 2023-08-18

**Authors:** Chukwuma Mbaeyi, Shahzad Baig, Rana Muhammad Safdar, Zainul Khan, Hamish Young, Jaume Jorba, Zubair M. Wadood, Hamid Jafari, Muhammad Masroor Alam, Richard Franka

**Affiliations:** ^1^Global Immunization Division, Center for Global Health, CDC; ^2^National Emergency Operation Center, Islamabad, Pakistan; ^3^World Health Organization, Islamabad, Pakistan; ^4^UNICEF, Islamabad, Pakistan; ^5^Division of Viral Diseases, National Center for Immunization and Respiratory Diseases, CDC; ^6^Polio Eradication Department, World Health Organization, Geneva, Switzerland; ^7^World Health Organization, Amman, Jordan.

**Summary**

**What is already known about this topic?**

Transmission of wild poliovirus type 1 (WPV1) has never been interrupted in Pakistan, one of two countries with ongoing endemic transmission.

**What is added by this report?**

Twenty WPV1 cases were reported in Pakistan during 2022, and one case during 2023 (as of June 2023), all clustered within a small geographic area in the southern region of Khyber Pakhtunkhwa province, an area with considerable security challenges and a history of vaccine hesitancy. Recent isolation of WPV1 from sewage in Karachi suggests surveillance gaps and improvements needed in immunization campaign quality.

**What are the implications for public health practice?**

To interrupt WPV1 circulation, the Pakistan polio program needs to meticulously track and sustain innovative efforts to vaccinate children who are regularly missed during polio vaccination activities, especially in reservoir areas affected by conflict and insecurity.

## Abstract

Since the establishment of the Global Polio Eradication Initiative in 1988, Pakistan remains one of only two countries (along with Afghanistan) with continued endemic transmission of wild poliovirus (WPV). This report describes Pakistan’s progress toward polio eradication during January 2022–June 2023. During 2022, Pakistan reported 20 WPV type 1 (WPV1) cases, all of which occurred within a small geographic area encompassing three districts in south Khyber Pakhtunkhwa. As of June 23, only a single WPV1 case from Bannu district in Khyber Pakhtunkhwa province has been reported in 2023, compared with 13 cases during the same period in 2022. In addition, 11 WPV1 isolates have been reported from various environmental surveillance (ES) sewage sampling sites to date in 2023, including in Karachi, the capital of the southern province of Sindh. Substantial gaps remain in the quality of supplementary immunization activities (SIAs), especially in poliovirus reservoir areas. Despite the attenuation and apparently limited geographic scope of poliovirus circulation in Pakistan, the isolation of WPV1 from an ES site in Karachi is cause for concern about the actual geographic limits of transmission. Interrupting WPV1 transmission will require meticulous tracking and sustained innovative efforts to vaccinate children who are regularly missed during SIAs and rapidly responding to any new WPV1 isolations.

## Introduction

Endemic transmission of indigenous wild poliovirus (WPV) type 1 (WPV1) has never been interrupted in Pakistan, which, along with Afghanistan, is one of two remaining countries where WPV1 remains endemic (*1*,*2*). Both countries share long borders with highly mobile populations and, as such, are considered a single epidemiologic block. The 2022–2026 Global Polio Eradication Initiative (GPEI) Strategic Plan’s stated goal of interrupting all WPV1 transmission worldwide by the end of 2023 (*3*) could be jeopardized by continued poliovirus circulation in Pakistan. This report describes Pakistan’s progress toward eliminating indigenous WPV1 transmission during January 2022–June 2023 and updates previous reports (*4*,*5*).

## Methods

Poliovirus surveillance data and vaccination campaign information were provided by the Pakistan National Emergency Operations Center and by other GPEI partners, including UNICEF and the World Health Organization (WHO). Weekly polio surveillance reports from the country and regional teams, as well as vaccination campaign reports shared by the country team were reviewed, as were national and subnational presentations prepared by the Pakistan polio program and immunization coverage surveys sponsored by Gavi, the Vaccine Alliance (conducted by a third party). Genomic sequencing analysis results were reviewed to ascertain the genetic relationship among polioviruses identified in WPV1 patients’ specimens and environmental sewage samples. A descriptive analysis of WPV1 patient characteristics, including age and essential immunization status, was conducted using Microsoft Excel.

## Results

### Immunization Activities

**Essential (routine) immunization.** For 2021, WHO and UNICEF estimated Pakistan’s national coverage with 3 doses of oral poliovirus vaccine (OPV) and 1 dose of inactivated poliovirus vaccine (IPV) by age 12 months at 83% for each vaccine (*6*). A 2021 third-party survey sponsored by Gavi, the Vaccine Alliance, indicated that the percentage of children aged 12–23 months who had received 3 OPV doses ranged (by province) from 45.1% in Balochistan to 94.9% in Punjab. No districts in the provinces of Balochistan, Khyber Pakhtunkhwa, and Sindh achieved ≥80% coverage, compared with 31 (86%) of 36 districts in Punjab province.

**Supplementary immunization activities**. Following the declaration of eradication of WPV type 2 in 2015 (*3*), and the globally synchronized withdrawal of trivalent OPV (tOPV) (containing Sabin strain types 1, 2, and 3) by all OPV-using countries in 2016 (*7*), most polio supplementary immunization activities (SIAs)* in Pakistan have been implemented using bivalent OPV (bOPV) (containing Sabin strain types 1 and 3). In response to circulating vaccine-derived poliovirus (cVDPV) type 2 (cVDPV2)^†^ outbreaks during 2019–2021, SIAs were implemented with tOPV and monovalent OPV type 2. During 2022, two national immunization days (NIDs) and six subnational immunization days (SNIDs) were conducted using bOPV. NIDs in Pakistan typically target approximately 44 million children aged <5 years, whereas SNIDs target smaller populations, depending on the areas identified by ongoing risk assessments. In addition, bOPV case-response vaccination activities were implemented following WPV1 isolation in south Khyber Pakhtunkhwa during March, April, and June 2022. Fractional-dose IPV (dose-sparing intradermal administration of IPV using one fifth of the regular intramuscular dose) was administered to eligible children along with OPV in south Khyber Pakhtunkhwa in SIAs that took place during June and August 2022. To date in 2023, one NID was conducted in January, and four SNIDs were conducted in February, March, May, and June.

Approximately 1.1 million vaccine-eligible children aged <5 years reside in the seven districts of south Khyber Pakhtunkhwa. Approximately 50,000 children in the region are regularly missed during OPV SIAs, including 19,500 children in the Mehsud tribal area of South Waziristan, where local health workers have been intimidated and prevented from vaccinating eligible children by militants since August 2022. The program has further been battling repeated boycotts by some communities during SIAs for reasons mostly unrelated to polio, such as requests for electricity services. Lot quality assurance sampling^§^ surveys, which assess SIA quality, continue to indicate substantial quality gaps in districts of south Khyber Pakhtunkhwa. Based on a 90% pass threshold, and surveyed using finger-marking, wherein a child’s fingernail is marked with indelible ink by vaccinators as a program indicator of receipt of OPV, the proportion of union councils (the lowest governmental administrative level) in south Khyber Pakhtunkhwa that reached the threshold ranged from 56% to 80% for SIAs conducted during August 2022–February 2023. Nationally, an estimated 505,750 eligible children were missed during NIDs in January 2023, including 22,466 (4%) refusals. In some areas, SIA quality assessments could potentially overestimate the actual proportion of children vaccinated because of the practice of fake finger-marking, wherein a child’s fingernail is marked by the vaccination team even though the child was not actually vaccinated.

### Poliovirus Surveillance

**Acute flaccid paralysis surveillance.** Pakistan reported a national nonpolio acute flaccid paralysis (NPAFP)^¶^ rate of 18.9 cases per 100,000 persons aged <15 years in 2022 (Table); provincial rates ranged from 10.7 to 28.6, exceeding the recommended surveillance sensitivity benchmark of ≥2 cases per 100,000 persons aged <15 years. As of May 16, 2023, the annualized 2023 national NPAFP rate is 15.3. Stool specimen adequacy** during 2022 and 2023 exceeded the target ≥80% indicator nationally and in all provinces. District-level performance indicators also generally improved compared with those in previous years. For example, the NPAFP rate in seven districts of south Khyber Pakhtunkhwa improved from 17.7 in 2021 to 24.9 in 2022. The program undertook internal reviews across all provinces and continues to implement surveillance-strengthening plans.

**TABLE:** Acute flaccid paralysis surveillance indicators and number of wild poliovirus and circulating vaccine-derived poliovirus cases reported, by province and surveillance period — Pakistan, January 2022–June 2023

Region	AFP surveillance indicators				No. of poliovirus cases							
	No. of AFP cases (nonpolio AFP rate)*		Adequate stool specimens, %^†^		Reported WPV1 cases				Reported cVDPV2 cases			
	2022	2023^§^	2022	2023	Jan–Jun 2022	Jul–Dec 2022	Jan–Jun 2023	Total	Jan–Jun 2022	Jul–Dec 2022	Jan–Jun 2023	Total
Azad Jammu and Kashmir	500 (26.4)	128 (15.5)	90.6	89.1	0	0	0	**0**	0	0	0	**0**
Gilgit-Baltistan	170 (24.9)	52 (17.4)	85.3	82.7	0	0	0	**0**	0	0	0	**0**
Islamabad	287 (28.6)	89 (31.9)	83.3	85.4	0	0	0	**0**	0	0	0	**0**
Khyber Pakhtunkhwa	4,659 (23.4)	1,258 (16.4)	83.7	87.3	14	6	1	**21**	0	0	0	**0**
Punjab	9,474 (18.3)	2,668 (15.5)	86.4	86.9	0	0	0	**0**	0	0	0	**0**
Balochistan	637 (10.7)	192 (8.0)	83.8	89.1	0	0	0	**0**	0	0	0	**0**
Sindh	3,300 (14.6)	1,104 (13.1)	83.8	87.3	0	0	0	**0**	0	0	0	**0**
**Total**	**19,027 (18.9)**	**5,491 (15.3)**	**85.2**	**87.1**	**14**	**6**	**1**	**21**	**0**	**0**	**0**	**0**

**Abbreviations**: AFP = acute flaccid paralysis; cVDPV2 = circulating vaccine-derived poliovirus type 2; WHO = World Health Organization; WPV1 = wild poliovirus type 1.

* Nonpolio AFP cases per 100,000 persons aged <15 years.

^†^ Stool specimens are considered adequate if two specimens are collected ≥24 hours apart within 14 days of paralysis onset and arrive at a WHO-accredited laboratory with reverse cold chain maintained and without leakage or desiccation. The standard WHO stool specimen indicator target is adequate stool specimen collection from ≥80% of AFP cases.

^§^ Annualized.

**Environmental surveillance.** A network of 114 environmental surveillance (ES) sewage collection sites in 80 districts serves as an ancillary means for detecting poliovirus circulation. Sewage samples collected monthly at these sites are tested for polioviruses and other (nonpolio) enteroviruses. During 2022, 37 (4%) of 1,024 sewage samples tested positive for WPV1, compared with 65 (8%) of 846 samples in 2021. In 2023 to date, 11 (1%) of 1,119 sewage samples have tested positive for WPV1, including samples from Lahore district in Punjab province (two); Dera Ismail Khan, Hangu, Peshawar, and South Waziristan districts in Khyber Pakhtunkhwa province (eight); and Karachi in Sindh province (one).

**FIGURE 1:**
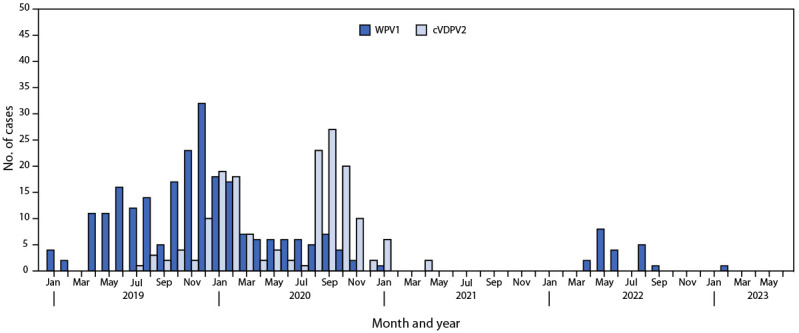
Wild poliovirus type 1 and circulating vaccine-derived poliovirus type 2 cases, by month — Pakistan, January 2019–June 2023 **Abbreviations**: cVDPV2 = circulating vaccine-derived poliovirus type 2; WPV1 = wild poliovirus type 1.

**FIGURE 2:**
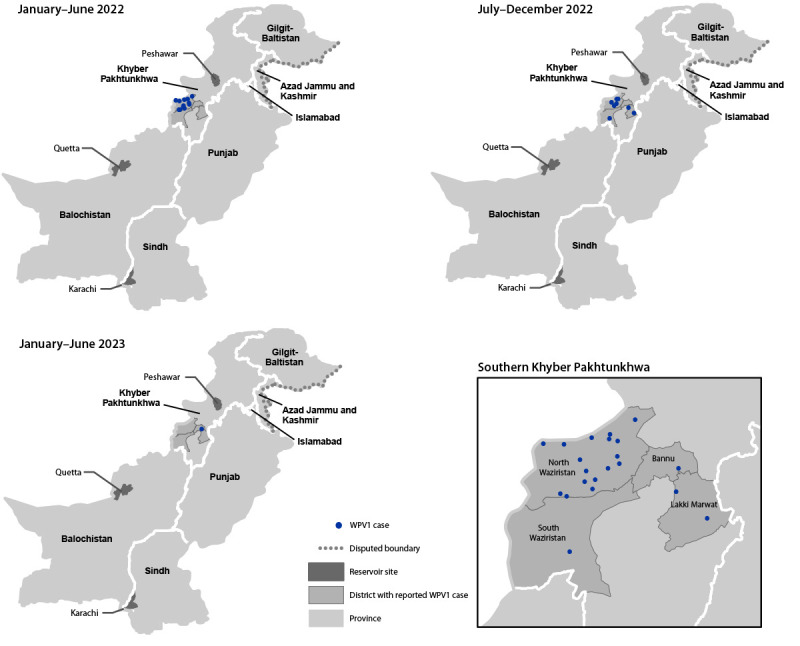
Location of cases of wild poliovirus type 1, by province and period — Pakistan, January 2022–June 2023 **Abbreviation**: WPV1 = wild poliovirus type 1.

**Epidemiology of poliovirus cases.** Twenty WPV1 cases were reported in Pakistan in 2022, compared with one case during 2021, 84 in 2020, and 147 in 2019 (Figure 1) (Figure 2) (*4*,*5*). As of June 23, 2023, a single WPV1 case has been reported in 2023. The case occurred in Bannu district, Khyber Pakhtunkhwa province, with paralysis onset on February 20, 2023 (Figure 2). All 20 WPV1 cases reported in 2022 occurred in three districts of south Khyber Pakhtunkhwa: North Waziristan (17), Lakki Marwat (two), and South Waziristan (one). Among the 21 WPV1 cases identified during the entire reporting period, patient ages ranged from 3 to 197 months (16 years) (median = 15 months); 17 (81%) had never received OPV through essential immunization; the remaining four (19%) had received 1–3 doses of OPV through essential immunization. No cVDPV2 has been reported in Pakistan since April 23, 2021, when the last of 165 cVDPV2 cases that occurred during July 2019–April 2021 was reported (Table) (Figure 1).

**Genomic sequence analysis of WPV1 isolates.** Analyses of the region coding the viral capsid protein VP1 indicated that the viruses of WPV1 cases all belong to a single genetic cluster sharing ≥95% sequence identity (the YB3C cluster). Among 11 environmental sample isolates for which sequencing results were available, three belonged to the YB3C cluster, which is endemic in Pakistan, whereas eight belonged to the YB3A cluster, currently circulating in eastern Afghanistan. The most recent isolation from Karachi also belonged to the YB3A cluster and differed by 5.3% in its VP1 coding region from its closest relative isolated from a sample collected in Karachi in January 2021. The level of deviation from its closest relative was much higher than the “orphan” virus criterion of ≥1.5%, indicating long-term undetected transmission of one lineage in Karachi missed by acute flaccid paralysis (AFP) surveillance and ES in the area.

## Discussion

The Pakistan polio program has made substantial progress toward the elimination of WPV1 transmission. The 21 WPV1 cases reported during January 2022–June 2023 represent a substantial reduction from the 84–147 WPV1 cases reported annually during 2019–2020 (*4*,*5*). Cases have been identified only in a small geographic area in south Khyber Pakhtunkhwa in districts afflicted by persistent insecurity and varying levels of community resistance. The genetic diversity of circulating WPV1 has narrowed from 10 clusters during 2019–2020 (*8*) to two indigenous clusters during the period under review.

Despite this progress, considerable obstacles to interrupting WPV1 transmission in Pakistan by the end of 2023 or the near future remain. AFP surveillance indicators have rebounded to or exceeded prepandemic levels nationally and provincially; however, continued isolation of WPV1 from ES sites in districts in south Khyber Pakhtunkhwa suggest ongoing gaps in AFP surveillance. WPV1 isolations from ES sampling sites in Lahore, Peshawar, and Hangu districts were genetically linked to WPV1 strains circulating in eastern Afghanistan, underscoring the ongoing risk for cross-border transmission as long as WPV1 circulation continues in Afghanistan. The “orphan” WPV1 ES isolate in Karachi highlights the current limitations of poliovirus surveillance and the challenges faced in reaching a substantial proportion of susceptible children in high-risk areas of Karachi.

To address these issues, meticulous microplanning of SIAs and systematic tracking of repeatedly missed children are needed, including among high-risk mobile populations moving across the shared border with Afghanistan. Wherever feasible, vaccination activities should be synchronized with Afghanistan in coordination with officials in that country and integrated with the delivery of other essential health services to gain the trust of hesitant communities. The safety and morale of frontline workers should remain a critical priority for the polio program, especially in light of occasional targeted attacks on polio workers and their accompanying security personnel.

### Limitations

The findings in this report are subject to at least one limitation. With refusals typically accounting for <5% of children missed for vaccination in most areas during polio SIAs, operational issues continue to account for the vast majority of continually missed children. A substantial limitation of this report is that estimates of vaccination coverage might be distorted by caregiver recall. Even when a given child’s finger is marked as evidence of vaccination during polio campaigns, the mark might not accurately reflect the true vaccination status because of the practice of fake finger-marking by some vaccinators.

### Implications for Public Health Practice

The 2021–2022 WPV1 outbreak in southeastern Africa linked to importation from Pakistan is apparently winding down (*9*); thus, the focus of GPEI partners remains on interrupting endemic WPV1 transmission in Pakistan and Afghanistan, as well as containing cVDPV outbreaks (*10*). Any new detection of poliovirus circulation in Pakistan would require an urgent response to facilitate prompt interruption of virus transmission. Halting the spread of WPV1 in Pakistan requires that the country maintain its strong commitment to ensuring that every child is reached, vaccinated, and protected from the debilitating effects of paralytic polio. 

## Footnotes

* SIAs are mass house-to-house vaccination campaigns targeting children aged <5 years with OPV, regardless of the child’s vaccination history.

† cVDPV, which can lead to paralysis, emerges as a result of attenuated OPV virus regaining neurovirulence after prolonged circulation in underimmunized populations.

§ Lot quality assurance sampling (LQAS) uses a small sample to assess the quality of vaccination activities after SIAs in union councils (referred to as “lots”). LQAS surveys seek evidence of vaccination (finger marking) by randomly selecting 60 children within each lot. If the number of unvaccinated children in the sample exceeds three, then the union council SIA is classified as having failed at a threshold of ≥90%, and additional vaccination activities in those areas are recommended. If the threshold of ≥90% (three or fewer unvaccinated children) is met, the union council SIA is classified as having passed.

¶ Acute flaccid paralysis (AFP) cases that are discarded as not having laboratory or other proof of poliovirus as the cause are considered NPAFP cases. The expected background rate of NPAFP illnesses is ≥2 per 100,000 persons aged <15 years per year, the standard WHO performance indicator target for sufficiently sensitive surveillance to detect a case of poliomyelitis.

** Stool specimens are considered adequate if two specimens are collected ≥24 hours apart within 14 days of paralysis onset and arrive at a WHO-accredited laboratory with reverse cold chain maintained and without leakage or desiccation. The standard WHO stool specimen indicator target is adequate stool specimen collection from ≥80% of AFP cases.
